# Supplementary material for: Are carbon emissions trading and green financial instruments synergistic? -Comprehensive quantitative research based on content analysis
Source: PLoS One. 2024 Mar 7;19(3):e0298601. doi: 10.1371/journal.pone.0298601 (PMC10919601; doi:10.1371/journal.pone.0298601)
Supplement: S1 Data — (ZIP) [file pone.0298601.s001.zip › Data/地级市Z_F工作报告.zip!/╔╜╬≈/┤≤═1⁄4╩╨/└┤╫╘╣1⁄2╓┌║┼í╛┬φ┐╦╩2╛▌═°í┐_2009.docx]

各位代表:

现在,我代表市人民政府向大会作工作报告,请予审议,并请市政协委员和其他列席会议的同志提出意见。

一、2008年工作回顾

2008年是本届政府履职的开局之年,也是很不平凡的一年。在市委的正确领导下,在市人大、市政协的监督支持下,全市人民认真落实科学发展观,迎难而上,奋力拼搏,同心同德,扎实工作,改革开放和现代化建设取得新的发展成就。

——经济发展有新增长。地区生产总值完成565.8亿元,比上年增长5.1%;固定资产投资276.5亿元,增长28.7%;财政总收入122.3亿元,增长31.4%;一般预算收入46.7亿元,增长27.1%;社会消费品零售总额232.6亿元,增长24.0%;城镇居民人均可支配收入13758元,增长19.6%;农民人均纯收入3354元,增长10.6%。

——转型发展有新进展。煤炭关小上大、兼并重组艰难前行。地方煤炭企业由270个关减到125个,同煤、中煤、山煤、煤运、国电等大型企业集团兼并重组地方煤炭企业、建设年产90万吨以上机械化矿井的计划积极推进。同煤塔山矿井生产能力达到1000万吨,同忻千万吨矿井建设顺利,东周窑千万吨矿井开工建设。塔山2×60万千瓦、国电2×66万千瓦,大唐2×30万千瓦竣工。大齿与中国重汽成功实现战略重组,中国重汽变速箱基地框架初露端倪。大同水泥集团百年湿法落后生产线停产,冀东年产500万吨现代化水泥生产线开工建设。中海油与同煤集团的煤基清洁能源项目、中华煤气与浑源的油母页岩开发项目正在顺利推进,引进大集团、大企业战略初见成效。

——城市建设有新跨越。强化城市规划龙头地位,邀请国际国内知名设计单位对御西、御东、口泉三大组团进行规划全覆盖,城市建设进入高水平规划指导下的新时代。城市基础设施建设强力推进,集中财力30亿元,展开了历史上规模空前的城市建设改造大决战。御河西路、迎宾街、向阳街、南三环、西三环等8条总长30多公里的城市道路,当年规划、当年设计、当年拆迁、当年开工、当年竣工。深化城市环境综合整治,共拆除各类违章和不协调建筑150多万平方米,完成城市绿化面积240万平方米。取缔小锅炉273个,新增集中供热面积600万平方米。市区20万户天然气置换全面完成,亏损和污染严重的煤气公司停产。二级以上良好天气达到307天。机械化清扫从零起步,垃圾电厂高标准建设,城市管理向科学化、规范化、标准化、精细化方向努力。城市规划、建设、管理三权分立,供热、供气、供水、污水处理集团化管理,保障基础巩固,保障机制创新,大规划、大建设、大城管的大格局体制形成,城市建设一年一个样的目标初步实现。

——文化产业有新举措。历史文化名城保护复兴工程启动,华严寺、善化寺、文庙、关帝庙、帝君庙、清真寺等修复工程顺利推进,华严寺广场、东小城、东城墙修复前期工程启动,3.28平方公里的核心保护区以及北至操场城街、南至新胜街、东至御河西路、西至新建路的环境保护区范围内,违法、无序等破坏性建设得以坚决纠正和清理。云冈大景区综合整治工程展开,云冈峪通道拆除不协调建筑50多万平方米,高标准绿化成为一道亮丽的风景线。拆除云冈公园与云冈景区分割的藩蓠,大景区建设迈出步伐。工人村、云冈、麻村、校尉屯等村庄的搬迁启动。恒山景区规划、设计、整治工程开始,悬空寺核心景区不协调建筑拆除,浑源历史文化街区保护工程全面推进。

——农村社会有新进步。农村经济总收入完成265.9亿元,增长9.7%;粮食总产14亿斤,增长41.6%。生态建设完成30万亩,高速公路环路绿带完成31.5公里。县乡公路改造477公里,村通水泥路1380公里。温室大棚等设施农业有新进展,孤山水库、文瀛湖、御河等前期工程到位,册田、恒山等22座水库除险加固工程国拨资金落实。全国最大的肉制品企业雨润集团百万头生猪屠宰项目落地,大龙头带动农业战略迈出步伐。

——民生问题有新改善。按照“拆迁谁、改变谁、造福谁”的民生政策,对百姓安居工程予以最大政策倾斜。投资20亿元,铺开民生安置工程100多万平方米,完成25万平方米,安置居民2355户。更加深情关注停产、破产企业职工安置问题,最大限度用活用足国家有关政策,在财力极其紧张的条件下挤出5个亿,用于解决停产、破产企业职工的养老、医疗、失业等保险费用,办理退休职工11916名,其中5289名职工享受提前退休政策。27万城乡困难群众享受低保政策3.5亿元,长期困扰大同的职工安置及社会稳定问题逐步开始解决。新建、改扩建中小学校52所,建筑面积37.4万平方米,大同一中新校、二中初中部、实验中学初中部开工建设,大同六中搬迁新校。城镇居民医疗保险试点启动,公共卫生体系进一步完善。体育事业进一步加强,竞技体育在奥运会上取得优异成绩。

——制度建设有新突破。规范土地出让制度,任何项目开发,必须通过招、拍、挂方式取得土地,禁止土地违法交易或变相违法交易。规范企业破产制度,纠正以重组之名、行国有资产流失之实的投机行为。规范税费征缴制度,打击偷逃税费的违法行为。规范规划建设管理制度,对违法建设实施严厉的控制和打击措施。规范人事管理制度,出台事业单位用人凡进必考的政策规定。建章立制,规范行为,以制度管人、管事、管权、管钱,无序的政府行为进入制度化的轨道,堵塞了国有资产、国土资源、税费征管、预算支出等四个方面流失的黑洞,制度收益突破20个亿,这是大同最重要、最基础、最管用的基本建设。政府自觉接受人民代表大会及其常委会的监督,接受人民政协的民主监督。全年共办理人大代表建议和政协提案496件,办复率100%。向市人大常委会提请审议地方性法规2件,制定政府规章2件。安全工作加强,安全责任制度进一步强化。依法治市有效推进,公民法制观念逐步增强。“平安大同”深入开展,社会治安形势好转。全面落实政府系统党风廉政建设责任制。煤焦领域反腐败斗争取得初步进展。

各位代表,过去一年,挑战非同寻常,成绩来之不易。大同取得的每一项进步,每一个变化,都凝聚着省委、省政府的深切关怀和中央、省直各部门的巨大支持,都是全市人民团结奋斗、共同努力的结果!在此,我代表市人民政府向全市人民,向人大代表、政协委员、各民主党派、人民团体和各界人士,向驻同解放军、武警官兵和公安干警,向所有关心支持大同改革发展的海内外各界朋友,表示衷心的感谢和崇高的敬意!

在肯定成绩的同时,也要清醒地看到,我们正面临前所未有的困难和挑战,特别是国际金融危机影响加深,部分行业和企业生产经营困难,经济增速下滑压力越来越大,长期制约我市经济健康发展的体制性、结构性矛盾更加突出。转型发展力度不够,招商引资突破不够,民营经济发展不够,民生问题解决不够。建设与管理不同步,重建轻管、管理滞后的问题仍然突出;拆迁与安置不同步,拆快建慢、百姓住房难、租房难的问题仍然突出;城市与乡村不同步,城乡差距拉大,三农问题仍然突出;期望与存在不同步,人民群众期望值高,但政府工作中的形式主义、官僚主义以及奢侈浪费现象仍然突出。这些都需在今后的工作中认真加以解决。

二、2009年工作目标和主要任务

当前,国际金融危机正在继续扩散和蔓延,涉及范围之广、影响程度之深、冲击力度之大,为百年所罕见。综观国际国内形势,今年将是进入新世纪以来我市发展最为困难的一年,保持经济平稳较快增长的任务异常艰巨。我们必须保持清醒的头脑,把困难估计得更严重一些,把应对考虑得更周密一些,把工作安排得更扎实一些。同时应当看到,我国仍处于重要战略机遇期,我国经济社会发展的基本面和长期向好的趋势没有改变。挑战与机遇并存,困难与希望同在。危机中蕴含的机遇要大于风险。机遇听之无声,望之无形,但却是最宝贵的创业机会,最稀缺的发展资源,最重要的跨越跳板。我们必须以等不得的紧迫感,输不起的危机感,坐不住的责任感,深刻地认识机遇,敏锐地发现机遇,果断地抓住机遇,创造性地用好机遇,强化转型发展,强化政策争取,强化招商引资,强化基础建设,为实现更高水平的又好又快发展打下坚实基础。

做好今年的政府工作,要以邓小平理论和“三个代表”重要思想为指导,深入贯彻落实科学发展观,把保持增长、促进发展作为首要任务,把结构调整、产业升级作为主攻方向,把抢抓机遇、争取投资作为重要抓手,把基础建设、城市创新作为战略重点,把扩大开放、招商引资作为根本途径,把改善民生、共享成果作为强大动力,在逆势中追赶前进,在困境中奋力崛起,努力夺取经济社会发展的新胜利,以优异成绩向新中国成立60周年献礼。

今年全市经济社会发展的主要目标是:地区生产总值增长8%,财政总收入增长8%,一般预算收入增长8%,全社会固定资产投资增长20%,社会消费品零售总额增长13%,城镇居民人均可支配收入增长12%,农民人均纯收入增长11%,人口自然增长率控制在6.2‰以内,城镇登记失业率控制在4%以内,万元GDP综合能耗下降6.5%,二氧化硫排放量控制在13.22万吨以下,化学需氧量排放量控制在5.14万吨以下。

实现上述目标,今年要着力做好以下工作。

(一)大力推进经济结构战略性调整,在转型升级中实现可持续发展

我市当前面临的困难,有全球金融危机与国内经济周期性调整叠加的外部原因,但最根本的还是自身结构性、素质性、体制性矛盾的集中反映。危机不过使短期困难与长期矛盾相互交织,使我们的“软肋”暴露得更加明显。面对经济增长的巨大压力,绝不能为了赢得一点速度,继续搞低水平扩张,粗放经营。要站在更高的历史起点上,以宏大的国际视野,制定具有国际水准、大同特色的产业发展目标。“不谋全局者不足以谋一域,不谋万世者不足以谋一时”。要忍得住暂时的阵痛,忍得住暂时的速度放缓,忍得住暂时的减收,忍得住暂时的压力,坚持科学发展不动摇,转型升级不畏难,苦练内功不浮躁。

煤炭是大同的基础产业。煤都,煤都,无煤何都之有?要继续强力支持同煤做大做强,塔山煤矿1500万吨达产达效,同忻1000万吨煤矿完成500万吨工作面,东周窑1000万吨完成35%建设进度,马道头1000万吨矿井完成前期工作,尽快实现同煤集团大同区域内产量突破1亿吨的目标。地方煤炭企业长期在“多、小、散、乱”的低水平上徘徊,矿井素质低、安全隐患大、资源浪费严重,已经走到“山穷水尽疑无路”的绝境。必须以壮士断腕、破釜沉舟的决心和勇气,在兼并重组、规模升级中杀出一条路。“凤凰涅槃”、“浴火重生”,方可走上“柳暗花明又一村”的坦途。要按照“总体规划、分步实施,重点突破、长短结合、成熟先行、整体提升”的原则,以单井年产量最低90万吨以上,企业规模不低于300万吨的建设标准,加快推进同煤、中煤、山煤、煤运、国电等大型企业集团对地方煤矿的兼并重组,关小上大,机械化、规模化、标准化开采,实现煤炭企业真正意义上的二次创业,使煤都建立在高素质、高水准、高效能和本质安全型矿井的基础之上。

煤都不能成为“卖煤”之都,要依托煤炭资源,延伸产业链,实现产业转型升级,能源基地整体创新。下大力推进同煤与中海油发展煤基清洁能源的合作。投资300亿元,建设年产40亿立方米煤制天然气,管道输送京津环渤海地区,实现国际一流综合型能源公司的战略目标。这是大同调整产业结构第一篇大文章,对大同未来发展具有重大历史意义。必须紧抓不放,全力推进,尽快落地。加快推进中华煤气公司与浑源合作的500万吨油母页岩转化常规油项目,总投资32亿元,年产100万吨油,25万吨液化气,350万吨半焦,销售突破50亿元。继续推进煤电一体化战略,国电三期2×66万千瓦空冷机组、大唐二期2×30万千瓦供热机组尽快发电,国投2×13.5万千瓦矸石电厂、左云二期、新荣、广灵、灵丘风电项目加快建设进度,御东热电2×30万千瓦、大热30万千瓦力争年内开工,国电2×100万千瓦、山西国际2×100万千瓦、同煤塔山2×66万千瓦要抓紧做好前期工作。冀东水泥两条日产4500吨熟料生产线力争年底建成投产。山橡集团3万吨氯丁橡胶力争前半年投产,二期3万吨项目尽快启动。

装备制造业是大同产业结构战略性调整的希望所在,打造“制造业之都”是大同几代人的梦想。要抓住中国重汽兼并重组大齿、进入大同的机遇,创造优良环境和优惠政策,提供更大的舞台和空间,让中国重汽在大同做更多、更大的文章。近期要抓好四大重要基地建设,即零部件出口基地、变速箱研发生产基地、齿轮铸锻基地、汽车车桥齿轮生产基地,使大齿成为中国齿轮行业的领先企业。要积极创造条件,推动大齿园区搬迁、产能扩张,发展重型专用汽车,实现销售收入每年增长100%,尽快突破100亿元的战略目标。要围绕建设全国轨道交通产业化基地的目标,做大做强同车公司,扩大具有国际一流水平电力机车的产能,向年销售突破100亿目标进军。加快山柴与德国MTU公司合资项目建设,确保年内投产,推进“军转民”的进程。充分利用万亩制造园区的空间和政策优势,吸引更多的制造业龙头企业入驻园区,推动大同制造有更大更快的发展。

医药和活性炭是大同的两个特色产业。要按照“总体规划,合理布局,整合重组,整体创新”的原则,提升医药和活性炭两个产业的市场竞争能力和综合实力。同药发展是个大战略。大同具有发展医药得天独厚的气候和地理条件,又具有人才、技术、市场、管理等产业基础,应当有一个更大的发展。今年要在制订同药发展战略的同时,实施原料药园区集中发展,威奇达药业、阿拉宾度生物公司要在搬迁重建中实现技术、环保、管理创新,产品升级,规模扩张,做大做强。积极支持普德药业、恒泰药业、新宝源药业等医药龙头企业、群体企业加速发展,培植医药工业的支柱地位。要推动同车公司建设国际一流水准的活性炭龙头企业,改变低水平重复建设、过度化竞争的格局,形成龙头带动,优势互补,互利共赢,良性发展产业群体,特而强,特而优,特而壮,成为市场竞争力强、盈利水平高的大产业。

经济困难期,也是调整转型期。在危机中抢抓机遇,信心比黄金更重要,责任比能力更重要,方向比努力更重要,速度比完美更重要。我们必须以前无古人的气魄和胆略,齐心协力做好结构调整这篇大文章,迎接产业振兴的新曙光。

(二)大力发展综合交通运输体系,在构建一小时经济圈中实现县域经济全面振兴

大同历史和现实的经验证明,经济要发展,交通须先行。高速公路就是致富路、发展路、黄金路。交通深刻地改变和影响一个地区的生产和生活方式,有力地推进经济社会的快速发展。长期以来,经济社会发展和人民群众日益增长的交通需求与交通运输远不能满足这种需求之间的矛盾,始终是大同面临的主要矛盾。大同经济社会发展相对落后,直接表现为交通发展滞后。建设便捷、畅通、安全、高效的综合交通运输体系,让人便于行,货畅其流,为商品和各种生产要素在全国范围内的自由流动、充分竞争提供快车道,是振兴大同的必由之路。

要抓住国家扩大内需的机遇,加快建设大同的高速公路网。今年4月底要确保大呼、荣乌两条高速公路开工,开辟大同到新荣、左云的快速通道,打通灵丘到浑源的高速连接。抓紧大浑、大马高速公路开工建设,实现大同到浑源、大同到阳高、天镇的高速通道。尽快做好浑广高速公路前期工作,力争明年年初开工建设,打通大同到浑源、广灵、河北蔚县的高速通道。推进高速公路东环外扩前期准备工作,尽快动工。大同境内六条高速公路,总长439公里,建成后将实现市、县、区高速公路全覆盖,形成市县一小时经济圈。同时,抓紧完成村村通水泥(油)路最后84个村、751公里的任务,年底实现“村村通”全覆盖。

要抓住国家建设城际高速铁路的机遇,开工建设大西(大同到太原、运城、西安)高速铁路,力争年底开工建设大张(张家口)高速铁路。三年建成后,大同对外交通就会形成一小时到达北京、太原,两小时到达西安的城际高速铁路圈。这对于强化大同交通枢纽地位、缩短与大城市的时空距离、融入环渤海经济圈、依托北京天津等特大城市发展自己,具有重大的现实意义和深远的历史意义。

抓好航空事业发展,完成机场跑道延伸、站坪扩建和候机楼改建,建设好大同的窗口和门户,展示现代大同的美好形象,让城市兴奋起来。

交通改变大同。三年后,大同内有高速公路一小时到达网,外有高速铁路一小时经济圈,天上有四通八达、飞向世界的航空线,将会放大三个效应。一是放大同城化效应。对外与北京、天津、西安、太原同城化,成为北京名副其实的“后花园”。对内与各县区同城化,强劲拉动县域经济大发展。二是放大走廊效应。交通走廊带来空间联系、产业关联、要素流动的便利性,形成交通走廊带动产业走廊、产业集聚发展的新格局。三是放大轴线扩散效应。交通网络贯穿、区域公共产品共享、产业链条延伸、资本逐利跨境连接、生产要素自由流动,增强了城市发展的吸引力,影响和带动周边区域经济发展。

规模如此宏大的交通建设,是大同几千年未有之盛事。交通建设,机遇在今天,决战在今天,发展在今天,翻身在今天。我们必须用我们的责任、效率、情感、服务,紧紧抓住历史的机遇,打好、打胜、打赢这场改变大同的战争。

(三)大力加强城市建设,在“一年一个样、三年大变样”的历史巨变中实现新大同崛起

城市是我们的家园。幸福家园有三个层次:宜居、利居、乐居。宜居是基础,利居是发展,乐居是幸福。让人乐居的城市,必然是景观优美、底蕴深厚、个性鲜明、温馨亲和的城市。山水人文景观让人愉悦,历史文化让人迷醉,个性特色让人记忆,亲和温暖让人留恋。能达到乐居这样最高境界的城市有两类:山水园林城市和历史文化名城。大同城市发展就是按照乐居城市的目标来规划布局的。御河的这一边是历史文化名城,御河的那一边是自然山水园林城市。在我们城市的大容器里,装的是两个大景观,一个是历史文化景观,一个是现代园林景观,一个传统,一个现代,这就产生了一种冲突,这种冲突就形成一种张力,这种张力就产生一种魅力,这种魅力就会震撼人的灵魂,给人带来深刻的思考和绚丽的想象。

继续推进名城保护复兴工程。今年完成华严寺、善化寺、清真寺、纯阳宫、文庙、关帝庙、帝君庙、法华寺等八大寺庙修复工程,完成东小城一期修复工程,基本完成华严寺广场、清真寺广场、鼓楼西街、东城墙修复工程,启动北城墙、代王府、北魏明堂遗址公园、北魏都城三大殿、鼓楼东街修复工程。让古城风貌的局部历史景观在文化艺术节期间初步展现出来。

加大新区建设力度。完成二中初中部、实验中学初中部、御东220千伏输变电工程建设;开工建设一医院、五医院、中医院、六医院、市职业技术学校、市卫校等卫生、教育工程;启动商业中心、商务中心、大剧院、博物馆、图书馆、美术馆建设工程,完成体育中心前期工作;基本完成雁同桥、兴云桥、南三环桥建设工程;完成雁同路东延、兴云街、平城东街、南环路、御河东路、文兴路、文瀛西路建设工程。

继续推进旧城改造工程。完成御苑路(同丰路)、前进街、红旗街、友谊街、新建路、永泰南路(工农路)、青年路、开源街、御西支路、龙园西路改造建设工程。积极推进矿区提升。重点完成南三环到同泉路延伸工程。这条总长近30公里的大道,将成为连接御东新区、平城老区、口泉矿区的发展大通道。今年城市道路建设总规模为21条路、近100公里,总投资50亿元。要按照建设城市副中心的规划要求,充分发挥同煤集团的主导建设力量,加快口泉区的开发建设,完善城市功能,提升城市品质。

城市建设要始终坚持生态立市、环境优先的指导思想。良好的生态环境是城市最重要的资源和资本,是城市的品牌和优势。在城市建设中,要下大力做好“水”文章和“绿”文章。御东要做好文瀛湖和御河两篇水文章,今年开工建设文瀛湖、御河防渗工程,文瀛湖蓄水要达到1000万立方米,面积6平方公里,使御东新区真正成为滨湖城市。御西和口泉区要治理好十里河、口泉河,截污蓄水,碧水长流。要把绿化大同作为基本政策,长期坚持下去。结合城市建设搞好道路绿化、环城绿化、环湖绿化、河道绿化、云冈峪绿化、智家堡垃圾场治理500亩绿化、一电厂储灰场350亩绿化、口泉千亩苗圃生态园林化,市区绿化总面积要突破1万亩。要大力推进城市环保工程。垃圾发电厂力争8月份运行,配套完成4个垃圾中转站。完成集中供热主管网延伸150公里,新增集中供热面积840万平方米,基本实现集中供热全覆盖;扩建东、西郊和开发区污水处理厂;全面改造城市下水管网,实现雨污分流;更新燃气公交车200辆;开工孤山水库城市应急水源、引黄供水管网、水厂建设工程。借鉴北京东城区管理模式,推进城市网格化管理。提升环卫工作水平,实现清扫机械化、收集容器化、清运密闭化、保洁全天化、管理现代化。

没有历史文化的城市没有底气,没有山水园林的城市没有灵气,没有个性魅力的城市没有人气。城市建设运动是一个需要千百万人参与的社会运动。人民群众是城市文明的基础,人人都是城市的美容师。一个美好的城市是建筑在先进市民文化理性自觉基础之上的。我们要把大同建设成为声名远播的文明和精神的高地,需要爱城如家、文明守法的市民精神。人们因城市的美丽而微笑,城市因人们的微笑而更加美丽。

(四)大力发展文化旅游大产业,在三大品牌打造中实现新跨越

文化是人类的灵魂,文化力是软实力的核心,文化力永远是一个城市赖以生存和发展的核心竞争力。胡锦涛同志指出:“一部人类社会发展史,是人类生命繁衍、财富创造的物质文明发展史,更是人类文化积累、文明传承的精神文明发展史。人类社会每一次跃进,人类文明每一次升华,无不镌刻着文化进步的烙印。”一个城市是有历史的,成熟的历史是由两种方式来记录的,一种是文字记录,一种是由它的建筑来记录。一个没有历史遗存的城市,虽然有过辉煌的历史,曾经发生过威武雄壮的历史事件,但它仍然是浅薄的。所以,历史文化遗存是不可再生、不可替代的第一珍贵资源。继续推进以中国历史文化名城为中心,以世界文化遗产云冈石窟、国家风景名胜区北岳恒山为两翼的文化旅游大产业,是大同长期坚持、一以贯之的大战略。

历史文化复兴工程核心是做好“老”的文章。老就是资格,老就是资本,老就是历史,老就是地位。历史不可以复制。满脸是沟壑纵横的皱纹,这是沧桑岁月留下的年轮;浑身是斑斑驳驳的伤疤,这是漫长风雨留下的印痕。修旧如旧、原汁原味,是文化遗存保护复兴的灵魂。名城复兴要精心打造“皇城气象”,关注北魏王朝在历史星河中的大历程、大路径、大脚印,梦回平城,再现辉煌。

世界文化遗产云冈大景区建设,今年要基本完成。工程内容包括云冈等五个村庄搬迁、北魏“山堂水殿”景观建设、北魏博物馆及演艺中心建设、游客服务中心、云冈研究院办公中心外迁、停车场及商业、餐馆配套服务体系建设、核心景区范围内的综合整治拆迁、旅游标牌、标识、家具、灯光等形象设计与建设、339国道改线等十八项工程,总投资10亿元。在这么短时间内完成如此浩大的工程,是一次挑战自我、挑战极限的冲刺,是政府向人民代表的庄严承诺,必须用生命和责任来担当。

恒山风景名胜大景区整治要全面铺开,今年要完成大景区入口广场、停车场及游客服务中心建设,悬空寺至恒山步行系统建设、岳门湾景区建设、新空中缆车系统建设,启动神溪湿地配套工程,文庙、永安寺、圆觉寺、县衙、民居等历史街区建设初见成效。进一步提高国家风景名胜区的自然度、美誉度、灵感度,还锦绣河山之美好形象,复兴中华山水文化之精神气象。

要精心打造“中国雕塑之都”的品牌形象,实施新云冈计划,将规划、设计、保护、创作、研究、储存、出版、宣传、管理融为一体,系统做好雕塑之都的基本建设。要聘请国内外知名专家组成城市雕塑指导委员会,精心策划雕塑竞赛、雕塑创作活动月、雕塑论坛等文化创意活动,积极筹备大同国际雕塑艺术节,将大同雕塑这张撼人心肺的文化名片推向世界。

要加大非物质文化遗产发掘、创作、策划、提升的力度,在名城建设剪纸、绢人、泥人、铜器、折纸、木雕等民间艺术博物馆,前馆后坊,深度展示民间艺术创作的审美过程。要发掘、整理、提升地方特色戏剧,将耍孩儿、罗罗腔、恒山道乐等非物质文化遗产整理改造成人民群众喜闻乐见、特色明显的地方文化品牌,发展地方大戏台,全方位展示大同历史文化的神韵。

21世纪是文化争雄的世界。大同底蕴深厚的历史文化是我们理想的养分、精神的能量、文明的情愫、情怀的表达。我们要以敬畏之心、仰慕之情,让尘封凝固、饱经岁月的历史,找回鲜活生动的生命和体温,让历史创造的一个又一个意境,闪现的一个又一个亮点,给我们今天的文明进步带来一次又一次智慧的启迪。

(五)大力发展特色农业,在集中力量抓突破中实现农业产业化发展新格局

农业、农村、农民等“三农”问题,始终是我们工作的重中之重。今年农业和农村工作的基本指导思想,是以产业化发展为统领,以建设现代农业示范区为契机,以集中力量抓突破为基本方法,以项目建设为载体,着力抓好市场拉动、龙头带动、基地联动、项目驱动四件大事,创造农业和农村工作的新格局。

抓好市场拉动,推动产业化升级转型。农业产业化是以市场为导向、以科技为支撑、以效益为核心、以规模为载体的产业集合体。没有市场就没有产业化,没有强大的市场就没有强大的产业。市场容量有多大,产业规模就有多大。抓市场是发展农业产业化的第一要务。要积极引进中国企业和中国民营企业500强之一的江苏雨润集团,投资38亿元,占地1500亩,建设面积70万平方米的农副产品物流交易中心,发展现代农副产品物流新型业态,成为山西最大、全国一流的跨区域、特大型农副产品交易中心、展示拍卖中心、信息发布中心、价格形成中心、产品标准化中心、品质认证中心、检验检测中心等七大中心,打造“买全国、卖全国”的国际化大市场。一期工程投资15亿元,今年动工,明年建成投入使用。既抓有形市场,也要抓无形市场。县、乡各级政府要把市场建设抓在手上,向市场要信息、要技术、要效益,为农民搞好产前、产中、产后服务。

抓好龙头带动,推动产业化纵深发展。落实“五个一”推进机制,坚持一个项目、一名领导、一套班子、一个方案、一抓到底。下大力扶持夏进乳业、华晟果蔬等10户有发展潜力的领军龙头企业,带动基地150万亩、农户30万户的产业化发展。雨润集团投资2亿元建设的百万头生猪屠宰生产线要尽快开工,力争年底投入运营。农业产业化龙头企业,是农业产业化的领头雁,是农业先进生产力的代表,是解决小生产与大市场联结的重要纽带,必须下大力抓好,抓出成效。

抓好基地联动,推动产业化规模扩张。做大畜牧基地。猪、羊、牛养殖要向园区化、标准化、良种化、规模化的方向发展,生猪出栏量要实现100万头向200万头跨越,羊要由130万只达到200万只,肉牛由7万头发展到20万头。做大蔬菜基地。温室大棚蔬菜要达到20万亩,今年增加1万亩。露天蔬菜要由50万亩上升到80万亩,蔬菜总量突破100万亩。做大优质杂粮基地。莜麦、荞麦、小米、豆类等优质小杂粮,是健康绿色食品,具有很大的市场潜力,要发展到150万亩。做大干果中药材基地。杏仁、核桃等干果和中药材要做到100万亩。大同作为黄芪主产地,要搞好深度的产业规划,从种植、饮片加工、原料药、功能保健食品、药品到高端产品的研究开发,形成一个优化升级的产业链条,规模化、标准化、产业化的种植基地,打造地球上品质最好的黄芪大品牌,向年销售收入30亿元进军。

抓好项目驱动,推动产业化基础创新。今年我市农口各部门争取中央、省级投资7.63亿元,共45个项目,加上地方配套,总投资10.36亿元。要采取项目打捆,统一规划,集中力量,重点突破的办法,改变撒胡椒面式的分配方法,提高资金的使用效益。实施好国家京津风沙源治理、首都水资源保护、退耕还林、太行山绿化及省十大造林绿化工程,完成生态建设任务28万亩。实施兴水战略,新增水地5万亩,恢复改善水地10万亩,发展节水农业10万亩,水土流失治理面积30万亩,解决饮水不安全人口16万人。推进应急水源工程建设,抓好孤山、守口堡、唐河、王千庄4座水库的建设,抓好册田、孤峰山、恒山、十里河和赵家窑等22座大中小型水库的除险加固工作。做好全省第三架人工增雨飞机落户大同基地建设工程。

加快城乡发展一体化进程,大力发展农村公共事业,推进社会主义新农村建设。继续强化惠民政策的财政倾斜,不断加大支农惠农的政策力度。在两年内实现“五个全覆盖”,即具备条件的建制村通水泥(油)路全覆盖,中小学校舍安全改造全覆盖,县乡村三级卫生服务体系特别是村级卫生室全覆盖,村通广播电视全覆盖,农村安全饮水全覆盖。

农民是我们的衣食父母,农村是我们的生命之根。我们昨天都从农村走来,永远不能忘记脚下的起点。要牢记“吃百姓之饭,穿百姓之衣,莫道百姓可欺,自己也是百姓”的古训,深怀爱民之心,恪守为民之责,善谋富民之策,多办利民之事,让百姓得到更多的实惠,过上更加美好的生活。

(六)大力推进开放兴市战略,在激活招商引资中实现发展质量升级

大同地处内陆,不能为内陆意识所束缚,固步自封;位居中部,不能为中游心态所满足,甘居落后。要进一步解放思想,开放大门,放开手脚,吹响招商引资的号角,激活招商引资的动力,落实招商引资的责任。要谋划大项目,争取大投入,上马大工程,引进大集团。去年以来,我们引进名列中国水泥建材行业前茅的冀东水泥集团、引进国内重型汽车龙头老大中国重汽集团、引进中国肉食品加工龙头老大江苏雨润集团、引进香港煤气老大中华煤气公司、引进中国石油三巨头之一中海油集团,招大引强战略有了一个好的开端。今年要继续实施引进大集团、大企业战略,结合大同的资源优势、产业特色,划分战区,制订计划,瞄准目标,组织专门班子,强化推进机制,上门招商、蹲点招商、跟踪招商、情感招商,重点在装备制造、煤炭化工、啤酒饮料、铝镁合金、农副产品加工、文化旅游等六个方面求突破,确实提高招商引资的有效性、务实性和创造性。

强化企业在招商引资中的主体作用,充分发挥民营企业的能量和优势,动员全社会力量做好招商引资这篇大文章。民营企业家是大同的稀有资源、宝贵财富,是社会主义事业的优秀建设者。企业家的行为是高尚的社会行为,他们创造的价值应当肯定,获得的地位应当尊重,合法的财富应当保护。大同未来经济能否持久增长,很大程度取决于民营经济发展、取决于民营企业家是不是由寻租活动转向创造价值的活动。大同发展的动力在民营经济,潜力在民营经济,希望在民营经济。全社会要营造重商、亲商、富商、安商的浓厚氛围,为民营企业创造健康成长的沃土,成就事业的舞台。民营企业家要以更开阔的视野,冲破“宁当鸡头,不当凤尾”的小农经济思想束缚,更大步伐地走出去,更高水平地引进来,学先进、傍大户、走正路,更好更快发展自己。

市场经济就是环境经济,招商引资的优势就是环境优势。规模的发展要靠发达的产业,而产业的发展则要靠环境的吸引力。政府效率是创造环境的核心。要推行政府首问负责制、服务承诺制、限时办结制、失职追究制、无偿代办制,精简审批事项,简化审批程序,实施窗口服务,落实并联审批。政府审批事项要由目前的400多项压缩到200项以内,便民中心窗口授权服务要到位,切实提高政府办事效率。努力将大同打造成为创业最宽松、办事最便捷、服务最文明,低建设成本、低交易成本、低生产成本、低管理成本、低行政成本、低社会成本的“三最六低”地区,营造良好的人文生态。有宽松政策,有自由开放的环境,有优质良好的服务,有滋养文明的雨露阳光,一切有志于干一番事业者,都可以在大同找到天地和舞台。大同要成为投资者的热土,创业者的乐园,成功者的天堂。

招商引资,要做大产业集群,做强龙头企业,做优服务品牌,做精园区题材。园区是招商引资最重要的平台。要下大力做好开发区扩区升位,制造区高点起步,塔山区循环发展,龙泉区配套建设,形成大同招商引资的大环境、大空间、大舞台。栽起梧桐树,吸引凤凰来。

天下没有免费的午餐,人间没有不付出的成果。招商引资关键在真抓实干。一分辛苦,一分收获。不干,人不可立,家不可富,商不可来,市不可兴。只有谋事、干事的大气魄、大手笔,才能成就伟业,开创招商引资的大局面。

(七)大力推进民生建设工程,在惠民阳光下实现成果共享

天下之大,民生为要。民生连着民心,民心凝聚民力。抓民生是一种责任,一种追求,一种使命。越是在困难的形势下,越要重视民意,体察民情,关注民生,下大力解决好民生的迫切问题,真正做到发展为了人民,发展依靠人民,发展成果由人民共享。

坚持优先发展教育事业。落实好城乡免费义务教育政策,加大对中等职业学校和高等院校家庭经济困难学生的资助,确保人人享有平等的受教育机会。扩大教育规模,实现人口增长与教育需求的平衡。优化教育结构,大力发展职业教育。努力提高教育质量,坚决扭转教育质量下滑的趋势,满足人民群众对优质教育的需求。加快实施中小学校舍安全工程,实行严格的行政首长负责制,推进农村中小学标准化建设,把学校建成最安全、家长最放心的地方。优化教育布局,推进一卫二卫、一技二技、会计商业等中等专业和技校的合并重组。落实名城保护规划,继续加大中小学布局调整力度,启动大同五中、九中、十二中、十三中、华严中学迁建工程,推进大同一中、北岳中学、十六中改扩建等工程建设进度。

把扩大就业再就业工作放在经济社会发展的突出位置。面对当前严峻的经济形势,要实施更加积极的就业再就业政策,落实好各项扶持政策。重点做好下岗失业和关闭破产企业人员再就业工作,积极帮助零就业家庭和就业困难人员就业。改善就业结构,转变就业观念,增强创业意识,加大实施“创业就业工程”的力度。适应国家扩大内需的政策,依托重大投资项目拉动就业。充分发挥服务业、劳动密集型产业、中小企业、非公有制经济在吸纳就业中的作用。要特别重视高校毕业生就业问题。全面加强就业服务体系建设,提高公共就业服务质量和水平,建立全市统一规范的人力资源市场。

大力发展医疗卫生事业,积极推进医疗卫生体制改革。健全基层医疗卫生服务体系,三年完成城市社区卫生服务中心配套工程和乡镇卫生院达标,提升县级医院水平,巩固完善新型农村合作医疗制度。重视中医药事业,充分发挥中医药在防病治病中的重要作用。农业县区各建设两所相当于二级乙等中心卫生院。开工建设市危险废物集中处理中心,完成市第三人民医院外科大楼建设,加快一、五、六医院及中医院建设进度。

不断健全覆盖城乡的社会保障体系。在坚持国家统一的社会保障政策的前提下,结合我市的实际情况,对城镇个体工商户、灵活就业人员及到龄退休但未满规定缴费年限的城镇企业职工实行“前补后延”的政策,解决老有所养的问题。抓住我市全国事业单位养老保险试点城市的机会,加快推进事业单位一体化养老保险制度。做好城镇居民医疗保险工作,使城镇居民和城镇各类职工全部能够享受到基本医疗保险待遇,真正做到病有所医。要着重解决高危行业和农民工的工伤保险参保问题,实现高危行业全部参加工伤保险。实现失业保险市级统筹,提高我市失业保险的统筹层次。健全社会救助体系,重点完善城乡居民最低生活保障制度和五保户供养、灾害救助等救助制度。加大社会福利建设力度,做好对各类优抚对象的优待抚恤工作,支持残疾人事业发展,加强老龄工作。

加快推进保障性住房建设。推进300万平米城市廉租住房和140万平米矿工棚户区安置房工程建设。切实抓好城市拆迁户安置工作,加快安置房建设的进度,真正做到拆迁谁、改变谁、造福谁,实现城市发展与老百姓安居双赢。实施农村危房改造,加大解决农民住房困难问题的力度。

推动社会事业全面健康发展。加强文化建设,满足人民群众不断增长的、多样化的文化需求,完善公共文化服务体系建设,搞好县级文化馆、乡镇文化站和村文化活动室的建设。继续实施广播电视“村村通”工程,推进我市数字电视网络资源整合,确保有线电视网络健康发展。加强城乡公共体育设施建设,努力构建全民健身服务体系,广泛开展全民健身活动。坚持计划生育的基本国策,稳定低生育水平,提高出生人口素质。

安全工作关系到人民生命财产安全,关系到经济社会发展全局。要进一步完善大安全工作格局,强化安全生产和安全监管的主体责任,建立完善安全生产规章制度,加强安全生产的专项整治,强化食品、药品安全监管,努力推动全市安全形势的进一步好转。认真做好信访工作,把稳定的任务落实到基层。进一步加强公安基础设施建设,完善城市报警与监控系统,建立打防管控的长效机制,促进社会治安的进一步好转,增强人民群众的安全感。

“民之所忧,我之所思;民之所思,我之所行”。人民群众是我们的主人,是我们干事创业的根本动力。我们要牢记宗旨抓民生,满怀深情抓民生,凝聚智慧抓民生,集中精力抓民生,把民生呼声作为第一信号,把民生需要作为第一选择,把民生满意作为第一标准。

(八)大力建设服务型政府,在为民办事的实践中实现改革创新

本届政府受任于全球金融危机百年一遇的特殊时期,责任重大,使命艰巨。我们要深入学习实践科学发展观,把干事创业作为旗帜,理想目标作为动力,抢抓机遇作为使命,职业精神作为追求,制度创新作为支点,勇于负责作为品格,善于思考作为能力,团结协调作为保证。以为民作宗旨,诚信作品牌,效率作生命,勤政作基石,严格作标准,认真作灵魂,学习作动力,清廉作表率,务实作根本,工作作快乐。努力为民办事,力争有所作为。深化行政管理体制改革。进一步理顺、完善部门管理职能,扎实推进政府机构改革,努力形成权责一致、分工合理、决策科学、执行顺畅、监督有力的行政管理体制。继续推进民主科学决策,完善公众参与、专家论证和政府决策相结合的决策体系,推行重大决策和重要事项咨询、听证、公示、通报、信息查询等制度,做到问政于民、问需于民、问计于民、问策于民。要以提高办事效率为中心,大力推进行政效能建设。推行“零距离”指导、“零停留”办公、“零差错”办理、“零关系”办事、“零利益”服务,不让工作在任何一个环节延误,不让文件在任何一个环节积压,不让差错在任何一个环节发生,不让群众在任何一个环节受到冷落。

推进民主政治建设。认真执行人大及其常委会的各项决议、决定,依法向人大及其常委会报告工作,主动接受人大的法律监督和工作监督。自觉接受人民政协的民主监督,虚心听取政协委员的建议和意见。高度重视人民群众监督和新闻舆论监督。不断提高依法行政的能力,推进法治政府建设,努力做到有权必有责,用权受监督,侵权要赔偿,违法受追究。支持人民法院、检察院依法履行职责,支持工会、共青团、妇联等群团组织依法依章工作,参与社会管理和公共服务。推进政务公开,建设阳光政府。落实政务信息公开条例,保障人民群众的知情权、参与权、表达权、监督权,赢得人民群众的充分理解、广泛支持和积极参与。

不断改进工作作风。作风就是形象,就是力量,就是保证。一个地方的工作,成在干部作风,败也在干部作风。干部的形象,好在作风,坏也在作风。人民群众对干部的态度,敬佩的是作风,不满的也是作风。要下大力转变作风,提倡看到了、想到了,就马上办、立即办、当下办的执行力,召之即来、来之能办、办之能成的执行力,决策后无阻力、无障碍、无条件、无理由的执行力。大力推行“一线工作法”,做到决策在一线落实、问题在一线解决、创新在一线体现、成效在一线检验。发扬“三事精神”,做到事必躬亲,事无巨细,事不过夜。推行“工作成果倒逼法”,以目标倒逼进度,时间倒逼程序,社会倒逼部门,下级倒逼上级,督查倒逼落实。

抓好反腐倡廉建设。坚持标本兼治、综合治理、惩防并举、注重预防的方针,全面落实党风廉政建设责任制,扎实推进惩治和预防腐败体系建设。加强廉政监察、执法监察、效能监察和审计监督,特别是加强对“一把手”的监督,落实各项规章制度,凭制度用权、靠制度办事、以制度管人,确保权力在监督下实施、在阳光下运行。要加强党性修养,“不以善小而不为,不以恶小而为之”,珍重人格,珍爱声誉,珍惜形象,常修为政之德,积小德养大德。要耐得住清贫,抗得住诱惑,管得住小节,自觉做到不仁之事不为,不义之财不取,不正之风不染,不法之事不干。深入开展煤焦领域反腐败专项斗争,查处违法违纪案件。继续强化“管理立市”的理念,健全各项管理制度。加强对各级财政扩大内需和改善民生的资金管理,抓好重大工程、重点行业、重点领域和重要环节的监管。严厉打击偷逃税费的不法行为,堵塞公共收益流失的漏洞。坚持集中财力办大事,严格控制行政成本,压缩会议,简化接待,零基预算,勤俭节约,反对铺张浪费。加强公务员队伍建设,以廉洁、务实、高效的政府形象取信于民。

昨天已经过去,发生的事情已不可改变;明天尚未到来,将要发生的事情无法预测。今天就在眼前,我们必须努力把握。我们必须做的事,必须我们做,不能推给别人;我们今日应该做的事,必须今日做,不能推给明天。时光易逝,人生苦短,我们必须以只争朝夕、快马加鞭的精神,把时间视为速度,看作力量,比作金钱,等同生命,创造无负于时代、无愧于人民的新业绩,开创大同的新时代。

各位代表,大同的发展已翻开了崭新的一页,我们正站在新的历史起点上,困难和挑战考验着我们,责任和使命激励着我们,历史和现实注视着我们。让我们紧密团结在以胡锦涛同志为总书记的党中央周围,在省委、省政府和市委的坚强领导下,在迎接挑战中找机遇,在破解难题中求突破,在扎实工作中渡难关,在科学发展中谋崛起,为创造新大同更加美好的明天,实现富民强市的大目标而努力奋斗。
